# Association of endometrial cancer risk with hypertension- an updated meta-analysis of observational studies

**DOI:** 10.1038/s41598-024-76896-8

**Published:** 2024-10-22

**Authors:** Agnieszka Drab, Wiesław Kanadys, Maria Malm, Krystian Wdowiak, Joanna Dolar-Szczasny, Bartłomiej Barczyński

**Affiliations:** 1 Chair of Preclinical Science, Department of Medical Informatics and Statistics with e-Health Lab, Medical University of Lublin, K. Jaczewskiego 5 Street, 20-059 Lublin, Poland; 2 Specialistic Medical Center Czechow, Lublin, Poland; 3 Faculty of Medicine, Medical University of Lublin, Lublin, Poland; 4 Department of General and Pediatric Ophtalmology, Medical University of Lublin, 20-079, Lublin, Poland; 5 I Chair, Department of Oncological Gynaecology and Gynaecology, Medical University of Lublin, Lublin, Poland

**Keywords:** Endometrial cancer, Hypertension, Meta-analysis, Endometrial carcinoma, Gynecologic oncology, Gynaecological cancer, Epidemiology, Renovascular hypertension

## Abstract

Endometrial cancer is one of the most common gynaecological cancers in the developed countries. The aim of this study was to determine the impact of hypertension on endometrial cancer risk. Databases: PubMed, Embase and the Cochrane Library were searched from January 2000 to June 2024. We used DerSimonian-Laird random-effects model for analysis. Risk estimates were extracted by two authors and summarized using meta-analytic methods. A total of 26 observational studies with 207,502 endometrial cancer cases were included in the study. Overall meta-analysis demonstrates significant association between hypertension and endometrial cancer risk (RR = 1.37, 95% CI: 1.27–1.47, *p* < 0.001). Subgroup analysis of the risk of endometrial cancer shows statistically significant higher risk in patients with BMI ≥ 30 kg/m2, diabetics, women who had their first menstrual period at the age of 11 years or earlier, and who had never given birth. Findings of this comprehensive review and meta-analysis indicate that hypertension is associated with higher overall risk of endometrial cancer.

## Introduction

Hypertension is a significant contributor to illness and death on a global scale, and it is a well-established factor that increases the risk of coronary heart disease and stroke^1–3^. Indeed, globally, high systolic blood pressure accounted for 10,8 million deaths and 236,5 million disability-adjusted life-years (DALYs) in 2019^4^. Important risk factors for hypertension include: overweight and obesity^5,6^, low physical activity^7,8^, high alcohol consumption^9,10^, dietary factors^11–14^ and use of non-narcotic analgesics^15^.

Endometrial cancer ranks as the sixth most frequently diagnosed cancer in women and the 15th most prevalent cancer overall^16,17^. In 2020, there were over 417,000 newly reported cases, resulting in 97,000 fatalities attributed to this form of cancer^16,17^. Incidence rates exhibit a tenfold disparity across different regions worldwide, with the highest rates observed in North America, Europe and Australia, and the lowest rates primarily found in various African regions and South Central Asia^16,17^. The likelihood of a woman developing endometrial cancer during her lifetime stands at approximately 3%, with the average age of diagnosis being 61 years^18^. Several studies have shown that the risk of endometrial cancer increases with: older age, early menstruation, late menopause, obesity, family history of endometrial cancer, exposure to radiation, infertility, and prolonged use of estrogen in hormone therapy^19–24^.

The current understanding of the biological mechanism(s) that might account for the detrimental impact of hypertension on endometrial cancer risk remains unclear. Some researchers have proposed that prolonged hypertension could potentially disrupt angiogenesis through matrix metalloproteinases 2 and 9^25^. Moreover, some have noted an association between hypertension and insulin resistance and, consequently, with IGF-1, a factor linked to cell growth and the progression of neoplastic conditions^25^. There is also a suggestion that prolonged hypertension might result in cellular senescence and the suppression of apoptosis^20^.

A previous systematic review and meta-analysis was conducted by Aune et al.^20^. However, due to the emergence of several new publications and the inclusion of additional variables in adjusted models, we decided to develop an updated systematic review and meta-analysis to investigate the link between hypertension and the risk of endometrial cancer. Our goal is to provide a clearer understanding of the strength of this association, identify potential sources of heterogeneity and assess the influence of other potential risk factors. Secondary outcomes included analysis of the modifying factors of endometrial cancer occurrence, including: family history of cancers, obesity, diabetes, age at menarche, parity, hormone replacement therapy, oral contraceptive use and cigarette smoking.

## Materials and methods

This systematic review with meta-analysis was performed in accordance with the preferred reporting items for systematic reviews and meta-analyses guidelines (PRISMA 2020, http://www.prismastatement.org)^26^.

### Search strategy and selection criteria

We identified studies published from January 2000 up to the June 2024 by performing a comprehensive research in Pubmed (http://www.ncbi.nlm.nih.gov/pubmed/), Embase (http://www.embase.com/); and the Cochrane Library (https://www.cochranelibrary.com/). The search included only publications submitted in English. Papers were selected using the following keywords: “hypertension” or “blood pressure” or “high blood pressure”; and “endometrial cancer” or “endometrial carcinoma” or “endometrial adenocarcinoma” or “endometrial tumor” or “endometrial neoplasm” or “EC”; and “risk” or “incidence”.

Our meta-analysis included studies that met the following criteria:

- they presented original data from case–control or cohort studies;
- the articles were published from January 2000 up to June 2024;
- the exposure was hypertension and endometrial cancer incidence;
- the studies were written in the English language.

The major reasons for exclusion of studies were that:

- exposure was to elevated blood pressure (not hypertension),
- the studies were systematic reviews or meta-analyses,
- that the articles were case studies only or case reports;
- insufficient data was provided;
- survival from endometrial cancer was the outcome;
- the studies investigated mortality or prognosis of endometrial cancer;
- the articles were in the form of books, reviews, duplicates.

## Data extraction and quality assessment

Two researchers (A.D. and W.K.) independently reviewed all abstracts obtained from database searches for relevance. Furthermore, they manually checked references and articles for additional studies. All doubts related to the study were addressed by consulting with the third author (M.M.). The following data were taken out: first author, year of publication, country and region of publishing, study period, study design/characteristics, confounding factors and covariates used in adjustments, number cases and controls/participants, risk estimates.

The Newcastle-Ottawa Scale (NOS) was used to evaluate the quality of the studies included to our research^27^. NOS consists of three variables of quality. These are as follows: selection (4 points), outcome (3 points), comparability (2 points). A total score of 9 points represents the highest methodological quality. All studies included to our meta-analysis were scored from 5 to 9. The average of NOS points for all studies was 6.69.

### Statistical analysis

All statistical analyses were performed using the Medical package of the STATISTICA 13.3 program (StatSoft Polska, Kraków, Poland). We applied DerSimonian and Laird random-effects model for analysis. RRs were reported with 95% confidence intervals. The findings of meta- analysis were presented on a forest plot at a confidence level of 95%. Publication bias was evaluated via Egger’s test^28^ and Begg’s test^29^. We also checked the possibility of publication bias by visually symmetry of the funnel plot. The heterogeneity of the articles included was assessed using the I^2^ tool, with values of 0-30%, 31-60%, 61-75%, and 76-100% indicating low, moderate, substantial, and considerable heterogeneity, respectively. Subgroup analysis was performed for the following, when data was available: family history of cancers, obesity, diabetes, age at menarche, parity, hormone replacement therapy, oral contraceptive, cigarette smoking. A p-value less of 0.05 was considered to be statistically significant.

## Results

**Fig. 1:**
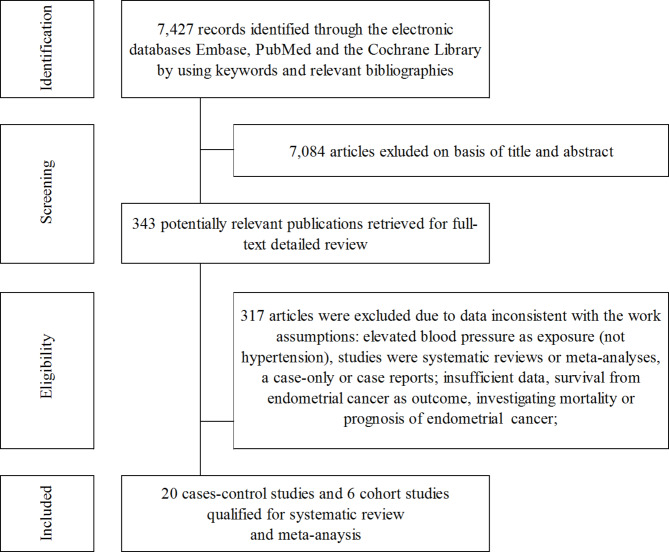
Flowchart of the selection procedure for studies included in the review and meta-analysis.

From searching the electronic databases, 7,427 articles were identified. In screening the papers, 7,084 articles were excluded on basis of the title or abstract. In addition, all duplicates were removed. This action, resulted in 343 potentially relevant articles. Two researchers (A.D. and W.D.) then screened these. This process led to exclusion of 317 articles (Fig. 1).

This meta-analysis evaluated the association between hypertension and endometrial cancer risk based on 20 case-control studies^30–49^, with a total of 8,938 cases and 41,265 controls, and 6 cohort studies with a total of 198,564 cases and 943,713 participants^50–55^. Of the 26 studies, 12 were conducted in the United States^30,33,34,39,42–45,51–53,55^, 7 in Europe [31,36,37,41,47,49,54], 3 in Asia^38,40,48^, 2 in Canada^32,35^, 1 in Mexico^46^ and one was international multicenter study^50^, (Table 1).

**Table 1:** Baseline characteristic of the included studies.

First author [References] Publication year	Location	Study period Years	No of Cases / Controls or Participants*	Age (Mean ± SD or Range)	Source of Subjects	NOS Score
**Case-control studies**						
Rodiguez [30] 2021	Texas USA	2012–2016	201 / 19,664	45–70+	PB	8
Kitson [31] 2018	England	2016–2017	150 / 746	75+	PB	7
Amankwah [32] 2013	Alberta Canada	2002–2006	467 / 1,030	59.0 ± 9.3	PB	7
Dallal [33] 2013	USA	2001–2004	62 / 124	67.5 ± 5.5	PBR	8
Torres [34] 2012	Minnesota USA	1985–2008	90 / 172	40–85	HBR	6
Friedenreich [35] 2011	Canada	2002–2006	515 / 962	30–79	PB	7
Rosato [36] 2011	Italy	1992–2006	454 / 798	18–79	HBR	6
Burbos [37] 2010	United Kingdom	2006–2009	149 / 2,898	59–72	PBR	5
Zhang [38] 2010	China	2004–2008	942 / 1,721	No data	HBR	8
Fortuny [39] 2009	New Jersey USA	2001–2005	469 / 467	33–88	PB	6
Reis [40] 2009	Istanbul Turkey	2002–2003	285 / 1,050	43–76	HBR	6
Luceteforte [41] 2007	Italy Switzerland	1988–2006	777 / 1,550	< 40–70+	PBR	8
Saltzman [42] 2007	USA	1985–1999	1,303 / 1,779	45–74	PBR	7
Strom [43] 2006	USA	1999–2002	511 / 1,412	50–79	PB	7
Soliman [44] 2006	Texas USA	2000–2004	117 / 238	25–88	HBR	6
Weiss [45] 2006	USA	1995–1999	1,281 / 1,704	45–74	PBR	5
Salazar-Martinez [46] 2000	Mexico	1995–1997	85 / 668	≤ 40–71+	HBR	5
Weiderpass [47] 2000	Sweden	1994–1995	709 / 3,368	50–74	PB	6
Niwa [48] 2000	Japan	1988–1997	134 / 376	40–70	HBR	7
Parslov [49] 2000	Denmark	1987–1994	237 / 538	27–49	PBR	5
**Cohort studies**						
Habeshian [50] 2024	Multicenter**	2009–2023	8273 / 30,609	Mean 63.3	HBR	9
Arthur [51] 2019	USA	2017	176 / 12,885	50–79	PB	7
Sponholtz [52] 2016	USA	1995–2013	274 / 47,577	21–69	PB	7
Ollbering [53] 2011	USA	1993/1996–2007	489 / 46,027	45–75	PB	8
Furberg [54] 2003	Norway	1974/1981–1996	130 / 24,460	20–49	PB	6
Folsom [55] 2003	USA	1986–2000	415 / 23,335	55–69	PB	7
Abbreviations: HBR, hospital-based registry; NOS, Newcastle-Ottawa Quality Assessment Scale; PB, population-based; PBR, population-based registry * number of controls for case-control studies; number of participants for cohort studies ** multicenter international study of Endometrial Cancer Consortium (E2C2)						

**Fig. 2:**
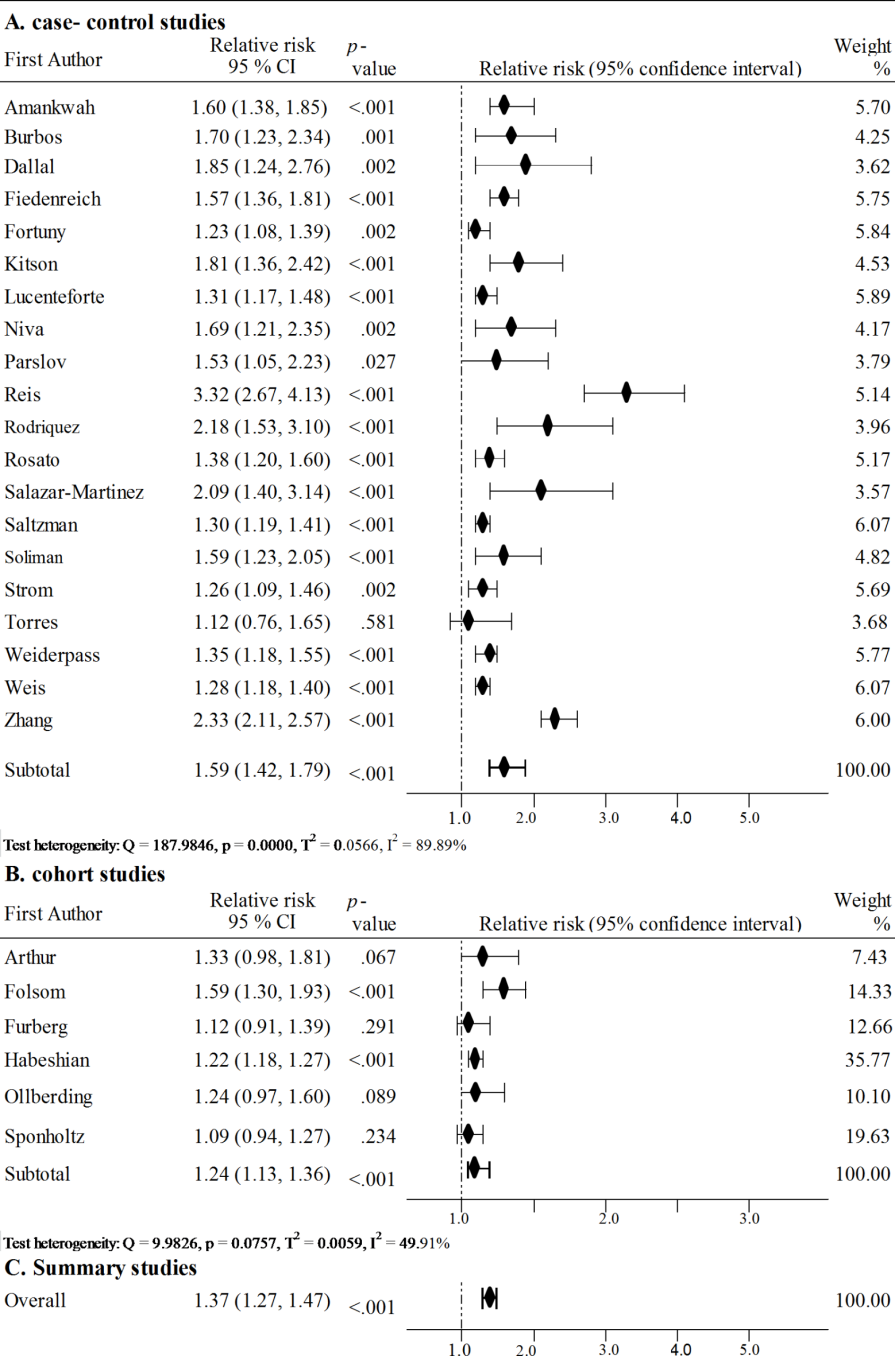
Forest plot of meta-analysis hypertension and endometrial cancer risk.

Figure 2 shows the results of a meta-analysis comparing the risk of endometrial cancer in patients diagnosed with hypertension to the risk of endometrial cancer in patients without hypertension divided into case-control and cohort studies. Accordingly, the risk of endometrial cancer in patients with hypertension is 1.37 times higher (95% CI: 1.27–1.47, *p* < 0.001). Moreover, the risk of endometrial cancer varies depending on the type of study (Z = 3.308, *p* = 0.001). In the case of case-control studies, this risk is 1.59 times higher (95% CI: 1.42–1.79, *p* < 0.001), while in cohort studies it is 1.24 times higher (95% CI: 1.13–1.36, *p* < 0.001). Moreover, out of 20 case-control studies, as many as 19 indicated a significantly higher risk of endometrial cancer in patients with hypertension. Only the results of the study by Torres^34^ were statistically insignificant (*p* = 0.581). The I^2^ value was 89.89%, which indicates high heterogeneity of studies, while the Egger’s test indicated no publication bias (b0 = 1.837, 95% CI: -1.284-4.957, t = 1.237, *p* = 0.232). Among the cohort studies, the results of two of them turned out to be statistically significant and indicated a higher risk of endometrial cancer in the presence of hypertension - Habeshian^50^ and Folsom^55^. Studies heterogeneity was moderate, with I^2^ = 49.91%, and Egger’s test did not indicate publication bias (b0 = 0.300, 95% CI: -2.267-2.867, t = 0.325, *p* = 0.762).

Due to the significant heterogeneity of the studies, an additional subgroup analysis was performed. Table 2 presents the results of the subgroup analysis. For as many as 8 out of 9 covariates, the outcomes turned out to be statistically significant. Hence, the risk of developing endometrial cancer is 2.14 times higher in patients with BMI ≥ 30 kg/m^2^ than in those with BMI ≤ 25 kg/m2 (95% CI: 1.86–2.46, *p* < 0.001), 1.86 times higher in patients with diabetes (95% CI: 1.65–2.09, *p* < 0.001), 1.41 times higher when using hormone replacement therapy (95% CI: 1.05–1.90, *p* = 0.023), 1.29 times higher in females whose menarche occurred at the age of 11 years or less (95% CI: 1.09–1.53, *p* = 0.003), and 1.26 times higher in patients who had never given birth (95% CI: 1.13–1.40, *p* < 0.001). Significantly lower risk of endometrial cancer occur among females using oral contraceptives (RR = 0.71, 95% CI: 0.63–0.79, *p* < 0.001), and patients who smoked cigarettes (RR = 0.80, 95% CI: 0.76–0.83, *p* < 0.001). In turn, family history of cancers (*p* = 0.052) did not significantly affect the risk of endometrial cancer.

**Table 2:** Modifying effects of other known-factors on risk of endometrial cancer.

Covariates	Variable	No. of studies	RR (95% CI)	*p*	I^2^
Family history of cancers	Yes	5	1.51 (1.00, 2.29)	0.052	93.49%
	No				
Obesity	BMI: ≥30 kg/m^2^	14	2.14 (1.86, 2.46)	< 0.001	89.87%
	BMI: ≤25 kg/m^2^				
Diabetes	Yes	15	1.86 (1.65, 2.09)	< 0.001	87.58%
	No				
Age at menarche	≤ 11 years	8	1.29 (1.09, 1.53)	0.003	92.63%
	> 11 years				
Parity	Nulliparous	11	1.26 (1.13, 1.40)	< 0.001	84.49%
	Gave birth				
Hormone replacement therapy	Used	5	1.41 (1.05, 1.90)	0.023	91.77%
	None used				
Oral contraceptive	Used	11	0.71 (0.63, 0.79)	< 0.001	88.85%
	None used				
Cigarette smoking	Yes	15	0.80 (0.76, 0.83)	< 0.001	32.41%
	No				

## Discussion

Our study is one of the few meta-analyses examining the relationship between hypertension and the development of endometrial cancer. Our findings suggest that hypertension is significantly associated with an increased risk of this cancer. However, it is important to note that we observed high heterogeneity, although Egger’s test indicated no publication bias.

Numerous studies in the past have investigated the link between hypertension and the development of endometrial cancer^30–49,56–63^. Almost all of them, except for the study conducted by Fortuny et al.^39^, indicated that hypertension increases the risk of this cancer. However, the results of these studies showed considerable variation. In case-control studies, the RR ranged from 1.10 to 6.34, while in cohort studies, it ranged from 1.02 to 1.90. These differences may be due to variations in study type, methodology, and the populations studied.

Our meta-analysis showed that hypertension increases the risk of endometrial cancer by 1.37 times (95% CI 1.27–1.47, *p* < 0,001), which aligns with the results of the only other meta-analysis on this topic, conducted by Aune et al.^20^, where RR = 1.61 (95% CI 1.41–1.85). When considering only case-control studies, we obtained an RR = 1.59 (95% CI 1.42–1.79), and for cohort studies, an RR = 1.24 (95% CI 1.13–1.36). In the Aune study^20^, these values were 1.73 (95% CI 1.45–2.06) and 1.32 (95% CI 1.12–1.56), respectively. It is worth noting that all RR values we obtained were lower than in the previous meta-analysis^20^, and the RR values from case-control studies in both meta-analyses were consistently higher than those from cohort studies. Since case-control studies are more susceptible to biases like recall and selection bias, it is notable that the association appeared stronger in such studies compared to cohort studies. However, it is important to highlight that a significant association was still observed in cohort studies, indicating that recall or selection biases alone may not fully account for the observed association^20^.

Hypertension is a proven risk factor for many cancers, and this list is likely to grow in the coming years^25^. It is also worth noting that numerous studies indicate that the presence of this condition worsens the prognosis for many cancers^64^. Hypertension may contribute to cancer development through several mechanisms, including remodeling of the extracellular matrix^25,65^, influencing the secretion of VEGF (Vascular Endothelial Growth Factor)^25,66^, generating ROS (Reactive Oxygen Species)^25^, and affecting the functioning of the RAA (Renin–Angiotensin–Aldosterone System)^67,68^ and MMPs (matrix metalloproteinases)^69^.

Remodeling of the extracellular matrix is triggered by inflammation related to hypertension^55^. This remodeling of blood vessel walls due to hypertension leads to ECM stiffening, disrupting the production of adhesive molecules that regulate cell-cell interactions, thereby accelerating tumor growth^55,65^.

VEGF plays a crucial role in both physiological and pathological angiogenesis by increasing blood vessel permeability and regulating endothelial cell proliferation^66^. Studies have shown that individuals with hypertension have elevated VEGF levels, leading to faster tumor progression and a poorer prognosis^66,70^.

Components of metabolic syndrome, including hypertension, contribute to cancer development mainly by increasing the production of ROS, estrogen (which promotes the development of endometrial cancer at high levels in the body), IGF-1, and adipokines^25^. It is also associated with excessive RAA system activity, with some studies indicating a connection between angiotensin II receptor gene mutations and cancer development^67,71^. MMPs are responsible for vascular changes typical of hypertension, and hypertension itself, through a yet-to-be-determined mechanism, leads to increased MMP-2 activation^72^. This, among other effects, disrupts cadherin structure, potentially impacting cancer development^72^. Increased MMP activity is also observed in many cancers, highlighting the need for further research into the link between hypertension and carcinogenesis via MMP activity modification, as this relationship is not fully understood^25^.

In the past, there have been speculations that certain antihypertensive drugs might influence cancer risk, as suggested by Fortuny et al.^39^, who linked the use of thiazide diuretics with an increased risk of endometrial cancer. However, more recent meta-analyses on this topic have shown that none of the currently used antihypertensive drugs increase the risk of any cancer^73^. It is also worth noting that in recent years, many publications have considered the potential anti-cancer effects of antihypertensive drugs^74^. Their role in treatment is linked either to their blood pressure-lowering effect (as seen with ACE inhibitors and ARBs) or to their pleiotropic effects (as observed with β-blockers and CCBs)^75,76^.

Our study demonstrated that obesity, diabetes, menarche before the age of 11, nulliparity, and hormone replacement therapy are factors that contribute to the development of endometrial cancer.

Obesity shares pathogenic mechanisms with hypertension, whose influence on cancer development has been discussed earlier^5^. Obesity also promotes the increased secretion of growth factors like leptin and insulin-like growth factor (IGF) pathways^77^, which, in turn, support cell growth and survival, thereby facilitating tumor progression^25^. It’s also important to note that in postmenopausal women, adipose tissue becomes one of the primary sources of estrogen in the body^78^, resulting in relatively high levels of these hormones in the blood for those who are obese^79^. Estrogens exhibit both mitogenic and mutagenic effects on the endometrium, promoting cancer development^79^.

Diabetes, especially type II, is widely recognized as a risk factor for endometrial cancer^80^. Hyperglycemia seems to promote cancer growth due to the high glucose demand of cancer cells^81^. Insulin resistance, a hallmark of type II diabetes, also appears to contribute to the development of endometrial cancer^82^. Excess insulin promotes the production of VEGF in the endometrium^80^, which accelerates tumor growth. This condition also increases endogenous estrogen levels in the blood due to reduced production of the SHBG protein^82^.

Early menarche leads to a longer duration of endometrial exposure to repeated menstrual cycles, with estrogens playing a key role in the pathogenesis of endometrial cancer^83^. Similarly, hormone replacement therapy also contributes to the risk of developing this cancer^84^. Pregnancy and breastfeeding reduce the number of menstrual cycles, making nulliparity, and to a lesser extent, infertility, factors that increase the risk of endometrial cancer^85^.

According to our analysis, the use of oral contraceptives and smoking cigarettes appear to reduce the risk of developing endometrial cancer. For women who do not use oral contraceptives, during the follicular phase of the menstrual cycle, there is a significant increase in the levels of estradiol and estrone, which is not counterbalanced by a corresponding rise in progesterone. This hormonal imbalance leads to substantial proliferation of endometrial cells^86^. In contrast, for women who use these contraceptives, the mitotic activity of endometrial cells is significantly lower. This reduction is primarily due to the presence of progestogen in these contraceptives, which decreases the expression of estrogen receptors in endometrial cells and increases the metabolic inactivation of estradiol in the body^86^.

The mechanism by which smoking affects the risk of endometrial cancer is not yet fully understood^87^. Current research suggests that smoking impacts the metabolism of endogenous estrogens more than their secretion^87^. Some researchers have also hypothesized a toxic effect of smoking on the ovaries. However, studies have shown conflicting results—some indicate that smoking leads to higher progesterone levels in the blood (which protects against endometrial cancer), while others suggest it results in lower progesterone levels (which promotes endometrial cancer). Due to these contradictory findings, further research is needed to better understand the molecular mechanism by which smoking may protect against endometrial cancer.

The current meta-analysis possesses several strengths that reinforce its findings, including a comprehensive and extensive search strategy, detailed subgroup and sensitivity analyses, and a large sample size, all of which contribute to the robustness of the link between hypertension and the risk of endometrial cancer.

However, this meta-analysis also has several limitations that should be taken into account when interpreting the results. The first limitation is that, in most studies on endometrial cancer risk, data regarding women’s use of estrogens and progestogens is often limited and sometimes contradictory. Only a few studies included in the meta-analysis provided sufficiently detailed information on patient management, including the use of these hormones. A second limitation is the significant heterogeneity among the included studies. Additionally, as with other meta-analyses, the possibility of publication bias exists; however, formal statistical tests indicated no evidence of such bias. Another limitation is the lack of data on menopausal status, which prevented us from conducting subgroup analyses based on this variable. Future research should consider potential risk factors for endometrial cancer beyond hypertension. It would also be essential to account for variables such as lifestyle factors (e.g., physical activity, diet, alcohol consumption, and smoking) and metabolic syndrome, as these influence hypertension and, consequently, may directly or indirectly affect the risk of developing endometrial cancer^20^. Additionally, studies examining the impact of hypertension on the risk of different types of endometrial cancer would be beneficial. Including the aforementioned data could reduce the heterogeneity of results, allowing for a more comprehensive analysis of the relationship between hypertension and the risk of endometrial cancer. Future studies incorporating genetic risk scores for hypertension could further clarify the causal relationship between hypertension and endometrial cancer^88^.

## Conclusions

Findings of this comprehensive review and meta-analysis indicate that women with hypertension may face a higher risk of developing endometrial cancer. Future research should aim to elucidate whether other factors influence this association, and explore the causal relationship behind the observed connection.

## Data Availability

The datasets used and analysed during the current study available from the corresponding author on reasonable request.

## References

[1] NCD Risk Factor Collaboration (NCD-RisC). Worldwide trends in hypertension prevalence and progress in treatment and control from 1990 to 2019: a pooled analysis of 1201 population-representative studies with 104 million participants. *Lancet*. **399** (10324), 520. 10.1016/S0140-6736(21)01330-1 (2022). 10.1016/S0140-6736(21)01330-1 PMC8446938 34450083

[2] Kario, K. et al. The WHO Global report 2023 on hypertension warning the emerging hypertension burden in globe and its treatment strategy. *Hypertens. Res.* **47**, 1099–1102. 10.1038/s41440-024-01622-w (2024). 38443614 10.1038/s41440-024-01622-w

[3] Boateng, E. B. & Ampofo, A. G. A glimpse into the future: modelling global prevalence of hypertension. *BMC public health*. **23**(1), 1906. (2023). 10.1186/s12889-023-16662-z 10.1186/s12889-023-16662-z PMC10546636 37789258

[4] GBD 2019 Risk Factors Collaborators. Global burden of 87 risk factors in 204 countries and territories, 1990–2019: a systematic analysis for the global burden of Disease Study. *Lancet*. **396** (10258), 1223–1249. 10.1016/S0140-6736(20)30752-2 (2020). 33069327 10.1016/S0140-6736(20)30752-2 PMC7566194

[5] Jia, G., Sowers, J. R. & Whaley-Connell, A. Obesity in hypertension: the role of the Expanding Waistline over the years and insights into the future. *Hypertension*. **81**(4), 687–690. 10.1161/hypertensionaha.123.21719 (2024). 38018438 10.1161/HYPERTENSIONAHA.123.21719 PMC10954419

[6] Parvanova, A., Reseghetti, E., Abbate, M. & Ruggenenti, P. Mechanisms and treatment of obesity-related hypertension-part 1: mechanisms. *Clin. Kidney J.* **17**(1), sfad282. 10.1093/ckj/sfad282 (2023). 38186879 10.1093/ckj/sfad282 PMC10768772

[7] Kazibwe, R. et al. Association between physical activity and clinical outcomes in high-risk hypertension: post-hoc analysis of SPRINT. *Am. J. Prev. Cardiol.* **16**, 100524. 10.1016/j.ajpc.2023.100524 (2023). 37576387 10.1016/j.ajpc.2023.100524 PMC10415631

[8] Shariful Islam, M. et al. Effect of leisure-time physical activity on blood pressure in people with hypertension: a systematic review and meta-analysis. *Sci. Rep.* **13**(1), 10639. 10.1038/s41598-023-37149-2 (2023). 37391436 10.1038/s41598-023-37149-2 PMC10313796

[9] Fuchs, F. D. & Fuchs, S. C. The Effect of Alcohol on blood pressure and hypertension. *Curr. Hypertens. Rep.* **23**(10), 42. 10.1007/s11906-021-01160-7 (2021). 34762198 10.1007/s11906-021-01160-7

[10] Algharably, E. A., Meinert, F., Januszewicz, A. & Kreutz, R. Understanding the impact of alcohol on blood pressure and hypertension: from moderate to excessive drinking. *Pol. Heart J. (Kardiologia Polska)*. **82**(1), 10–18. 10.33963/v.kp.98704 (2024). 10.33963/v.kp.98704 38230497

[11] Appel, L. J. The effects of Dietary factors on blood pressure. *Cardiol. Clin.* **35**(2), 197–212. 10.1016/j.ccl.2016.12.002 (2017). 28411894 10.1016/j.ccl.2016.12.002

[12] Altawili, A. A. et al. An exploration of dietary strategies for Hypertension Management. *Narrative Rev. Cureus*. **15**(12), e50130. 10.7759/cureus.50130 (2023). 10.7759/cureus.50130 PMC10771610 38186513

[13] Batubo, N. P., Moore, J. B. & Zulyniak, M. A. Dietary factors and hypertension risk in West Africa: a systematic review and meta-analysis of observational studies. J Hypertens. 1;41(9): 1376–1388. doi: (2023). 10.1097/HJH.0000000000003499 10.1097/HJH.0000000000003499 PMC10399948 37432889

[14] Evans, R. G. et al. Renal and dietary factors associated with hypertension in a setting of disadvantage in rural India. *J. Hum. Hypertens.* **35**(12), 1118–1128. 10.1038/s41371-020-00473-5 (2021). 33462389 10.1038/s41371-020-00473-5

[15] Rivasi, G. et al. The effects of Pain and analgesic medications on blood pressure. *Curr. Hypertens. Rep.* **24**(10), 385–394. 10.1007/s11906-022-01205-5 (2022). 35704141 10.1007/s11906-022-01205-5 PMC9509303

[16] Lortet-Tieulent, J., Ferlay, J., Bray, F. & Jemal, A. International patterns and trends in Endometrial Cancer incidence, 1978–2013. *J. Natl Cancer Inst.* **JNCI**(4), 354–361. 10.1093/jnci/djx214 (2017). 10.1093/jnci/djx214 29045681

[17] Sung, H. et al. Global Cancer statistics 2020: GLOBOCAN estimates of incidence and Mortality Worldwide for 36 cancers in 185 countries. *CA: Cancer J. Clin.* **71**(3), 209–249. 10.3322/caac.21660 (2021). 33538338 10.3322/caac.21660

[18] Gu, B. et al. Variations in incidence and mortality rates of endometrial cancer at the global, re-gional, and national levels, 1990–2019. Gynecologic Oncology 2021. 10.1016/j.ygyno.2021.01.036 10.1016/j.ygyno.2021.01.036 33551200

[19] Crosbie, E. J. et al. Endometrial cancer. *Lancet*. **399**(10333), 1412–1428. 10.1016/S0140-6736(22)00323-3 (2022). 35397864 10.1016/S0140-6736(22)00323-3

[20] Aune, D. et al. Anthropometric factors and endometrial cancer risk: a systematic review and dose–response meta-analysis of prospective studies. *Ann. Oncol.* **26**(8), 1635–1648. 10.1093/annonc/mdv142 (2015). 25791635 10.1093/annonc/mdv142

[21] Gupta, N. et al. Endometrial cancer risk factors, treatment, and survival outcomes as per the European Society for Medical Oncology (ESMO) - European Society of Gynaecological Oncology (ESGO) - European Society for Radiotherapy and Oncology (ESTRO) risk groups and International Federation of Gynecology and Obstetrics (FIGO) staging: an experience from developing world. *J. Cancer Res. Ther.* **19**(3), 701–707. 10.4103/jcrt.jcrt_1173_21 (2023). 37470597 10.4103/jcrt.jcrt_1173_21

[22] Naqvi, A. et al. The impact of obesity and bariatric surgery on the immune Mi-Croenvironment of the endometrium. *Int. J. Obes.* **46**(3), 605–612. 10.1038/s41366-021-01027-6 (2021). 10.1038/s41366-021-01027-6 PMC8872994 34857870

[23] Katagiri, R. et al. Reproductive Factors and Endometrial Cancer Risk Among Women. *JAMA network open* **6**(9), e2332296. 10.1001/jamanetworkopen.2023.32296 (2023). 10.1001/jamanetworkopen.2023.32296 PMC10481237 37669051

[24] Kitson, S. J. et al. Predicting risk of endometrial cancer in asymptomatic women (PRECISION): model development and external validation. *BJOG*. **131**(7), 996–1005. 10.1111/1471-0528.17729 (2024). 38073256 10.1111/1471-0528.17729

[25] Connaughton, M. & Dabagh, M. Association of Hypertension and Organ-Specific Cancer: a Meta-analysis. *Healthc. (Basel)*. **10**(6), 1074. 10.3390/healthcare10061074 (2022). 10.3390/healthcare10061074 PMC9222904 35742125

[26] Page, M. J. et al. The PRISMA 2020 statement: an updated guideline for reporting systematic reviews. *BMJ*. **372**, n71 (2021). 33782057 10.1136/bmj.n71 PMC8005924

[27] Ottawa Hospital Research Institute. The Newcastle-Ottawa Scale (NOS) for assessing the quality of nonrandom-ized studies in meta-analyses, (Online). ; (cited 25 February 2024; available from: URL (2011). http://www.ohri.ca/programs/clinical_epidemiology/oxford.asp.).

[28] Begg CB & MazumdarM Operating characteristics of a rank correlation test for publication bias. *Biometrics*. **50**, 1088–1101 (1994). 7786990

[29] Egger, M., Davey Smith, G., Schneider, M. & Minder, C. Bias in meta-analysis detected by a simple, graphical test. *BMJ*. **315**, 629–634 (1997). 9310563 10.1136/bmj.315.7109.629 PMC2127453

[30] Rodriguez, A. M. et al. Factors associated with endometrial cancer and hyperplasia among middle-aged and older hispanics. *Gynecol. Oncol.* **160**(1), 16–23. 10.1016/j.ygyno.2020.10.033 (2021). 33221024 10.1016/j.ygyno.2020.10.033 PMC8142520

[31] Kitson, S. J. et al. The unrecog-nized burden of cardiovascular risk factors in women newly diagnosed with endometrial cancer: A prospective case control study. *Gynecol Oncol*. **148**(1), 154–160. 10.1016/j.ygyno.2017.11.019. PMID: 29174567 (2018). 10.1016/j.ygyno.2017.11.019 PMC6562057 29174567

[32] Amankwah, E. K. et al. Anthropometric measures and the risk of endometrial cancer, overall and by tumor microsatellite status and histological subtype. *Am. J. Epidemiol.* **177**(12), 1378–1387. 10.1093/aje/kws434 (2013). 23673247 10.1093/aje/kws434 PMC3732018

[33] Dallal, C. M. et al. Obesity-related hormones and endometrial cancer among postmenopausal women: a nested case–control study within the BwFIT cohort. *Endocr. Relat. Cancer*. **20**(1), 151–160. 10.1530/ERC-12-0229 (2013). 23222000 10.1530/ERC-12-0229 PMC4038326

[34] Torres, M. L. et al. Risk factors for developing endometrial cancer after benign endometrial sampling. *Obstet. Gynecol.* **120**(5), 998–1004. 10.1097/aog.0b013e31826b9fef (2012). 23090515 10.1097/aog.0b013e31826b9fef PMC3711271

[35] Fiedenreich, C. et al. Case–control study of the metabolic syndrome and metabolic risk factors for endometrial cancer. *Epidemiol. Biomarkers Prev.* **20**(11), 2384–2395. 10.1158/1055-9965.EPI-11-0715 (2011). 10.1158/1055-9965.EPI-11-0715 21921255

[36] Rosato, V. et al. Metabolic syndrome and endometrial cancer risk. *Ann. Oncol.* **22**(4), 884–889. 10.1093/annonc/mdq464 (2011). 20937645 10.1093/annonc/mdq464

[37] Burbos, N. et al. Predicting the risk of endometrial cancer in postmenopausal women presenting with vaginal bleeding: the Norwich DEFAB risk assessment tool. *Br. J. Cancer*. **102**(8), 1201–1206. 10.1038/sj.bjc.6605620 (2010). 20354525 10.1038/sj.bjc.6605620 PMC2856001

[38] Zhang, Y. et al. The association between metabolic abnormality and endometrial cancer: a large case-control study in China. *Gynecol. Oncol.* **117**(1), 41–46. 10.1016/j.ygyno.2009.12.029 (2010). 20096921 10.1016/j.ygyno.2009.12.029

[39] Fortuny, J. et al. Risk of endometrial cancer in relation to medical conditions and medication use. *Cancer Epidemiol. Biomarkers Prev.* **18**(5), 1448–1456. 10.1158/1055-9965.EPI-08-0936 (2009). 19383893 10.1158/1055-9965.EPI-08-0936 PMC2763278

[40] Reis, N. & Beji, N. K. Risk factors for endometrial cancer in Turkish: results from a hospital-based case–control study. *Eur. J. Oncol. Nurs.* **13**(2), 122–127. 10.1016/j.ejon.2009.01.007 (2009). 19332387 10.1016/j.ejon.2009.01.007

[41] Lucenteforte, E. et al. Diabetes and endometrial cancer: effect modification by body weight, physical activity and hypertension. *Br. J. Cancer*. **97**(7), 995–998. 10.1038/sj.bjc.6603933 (2007). 17912243 10.1038/sj.bjc.6603933 PMC2360421

[42] Saltzman, B. S. et al. Diabetes and endometrial cancer: an evaluation of the modifying effects of other known risk factors. *Am. J. Epidemiol.* **167**(5), 607–614. 10.1093/aje/kwm333 (2008). 18071194 10.1093/aje/kwm333

[43] Strom, B. L. et al. Case-control study of postmenopausal hormone replacement therapy and endometrial cancer. *Am. J. Epidemiol.* **164**(8), 775–786. 10.1093/aje/kwj316 (2006). 16997897 10.1093/aje/kwj316

[44] Soliman, P. T. et al. Associa-tion between adiponectin, insulin resistance, and endometrial cancer. *Cancer*. **106**(11), 2376–2381. 10.1002/cncr.21866 (2006). 16639730 10.1002/cncr.21866

[45] Weiss, J. M. et al. Risk factors for the incidence of endometrial cancer according to the aggressiveness of disease. *Am. J. Epidemiol.* **164**(1), 56–62. 10.1093/aje/kwj152 (2006). 16675538 10.1093/aje/kwj152

[46] Salazar-Martínez, E. et al. Case-control study of diabetes, obesity, physical activity and risk of endometrial cancer among Mexican women. *Cancer Causes Control*. **11**(8), 707–711. 10.1023/a:1008913619107 (2000). 11065007 10.1023/a:1008913619107

[47] Weiderpass, E. et al. Body size in different periods of life, diabetes mellitus, hypertension, and risk of postmenopausal endometrial cancer (Sweden). *Cancer Causes Control*. **11**(2), 185–192. 10.1023/a:1008946825313 (2000). 10710204 10.1023/a:1008946825313

[48] Niwa, K. et al. A case-control study of uterine endometrial cancer of pre- and post-menopausal women. *Oncol. Rep.* **7**(1), 89–93 (2000). 10601598

[49] Parslov, M. et al. Risk factors among young women with endometrial cancer: a Danish case-control study. *Am. J. Obstet. Gynecol.* **182**(1), 23–29. 10.1016/s0002-9378(00)70486-8 (2000). 10649152 10.1016/s0002-9378(00)70486-8

[50] Habeshian, T. S. et al. Hypertension and risk of Endometrial Cancer: a pooled analysis in the epidemiology of Endometrial Cancer Consortium (E2C2). *Cancer Epidemiol. Biomarkers Prev.* **33**(6), 788–795. 10.1158/1055-9965.EPI-23-1444 (2024). 38530242 10.1158/1055-9965.EPI-23-1444 PMC11145161

[51] Arthur, R. S. et al. Metabolic syndrome and risk of endometrial cancer in postmenopausal women: a prospective study. *Cancer Causes Control*. **30**(4), 355–363. 10.1007/s10552-019-01139-5 (2019). 30788634 10.1007/s10552-019-01139-5 PMC6886235

[52] Sponholtz T. R. et al. Body Size, Metabolic Factors, and Risk of Endometrial Cancer in Black Women. *Am. J. Epidemiol.* **183**, 259–268. (2016). 10.1093/aje/kwv186 PMC4753280 26823438

[53] Ollberding N. J. et al. Legume, soy, tofu, and isoflavone intake and endometrial cancer risk in postmenopausal women in the multiethnic cohort study. *J Natl. Cancer Inst*. **104**, 67–76 (2012). 10.1093/jnci/djr475 PMC3250383 22158125

[54] Furberg, A. S. & Thune, I. Metabolic abnormalities (hypertension, hyperglycemia and overweight), lifestyle (high energy intake and physical inactivity) and endometrial cancer risk in a Norwegian cohort. *Int. J. Cancer*. **104**, 669–676 (2003). 12640672 10.1002/ijc.10974

[55] Folsom, A. R. et al. Glycemic index, glycemic load, and incidence of endometrial cancer: the Iowa women’s health study. *Nutr. Cancer.* **46**, 119–124 (2003). 10.1207/S15327914NC4602_03 14690786

[56] Wang Y. et al. Association between metabolic disorders and clinicopathologic features in endometrial cancer. Front Endocrinol (Lausanne) ; 15:1351982. 10.3389/fendo.2024.1351982 (2024). 10.3389/fendo.2024.1351982 PMC11385602 39257906

[57] Seretis, A. et al. Association between blood pressure and risk of cancer development: a systematic review and meta-analysis of observational studies. *Sci. Rep.* **9**(1), 8565. 10.1038/s41598-019-45014-4 (2019). 31189941 10.1038/s41598-019-45014-4 PMC6561976

[58] Inoue, M. et al. A case-control study on risk factors for uterine endometrial cancer in Japan. *Jpn J. Cancer Res.* **85**, 346–350 (1994). 8200846 10.1111/j.1349-7006.1994.tb02365.x PMC5919475

[59] Goodman, M. T. et al. Diet, body size, physical activity, and the risk of endometrial cancer. *Cancer Res.* **57**, 5077–5085 (1997). 9371506

[60] McCann, S. E. et al. Diet in the epidemiology of endometrial cancer in western New York (United States). *Cancer Causes Control*. **11**, 965–974 (2000). 11142531 10.1023/a:1026551309873

[61] Trabert, B. et al. Metabolic syndrome and risk of endometrial cancer in the United States: a study in the SEER-medicare linked database. *Cancer Epidemiol. Biomarkers Prev.* **24**, 261–267 (2015). 25587111 10.1158/1055-9965.EPI-14-0923 PMC4295647

[62] Shao, Y. et al. Insulin is an important risk factor of endometrial cancer among premenopausal women: a case-control study in China. *Tumour Biology: J. Int. Soc. Oncodevelopmental Biology Med.* **37**(4), 4721–4726. 10.1007/s13277-015-4229-x (2016). 10.1007/s13277-015-4229-x 26511973

[63] Zhao, J. et al. Risk factors of endometrial cancer in patients with endometrial hyperplasia: implication for clinical treatments. *BMC Women’s Health*. **21**(1). 10.1186/s12905-021-01452-964. (2021). 10.1186/s12905-021-01452-9 PMC8390278 34433451

[64] Teleka S. et al. Association between blood pressure and BMI with bladder cancer risk and mortality in 340,000 men in three Swedish cohorts. *Cancer Med.* 10, 1431–1438. 10.1002/cam4.3721 (2021). 10.1002/cam4.3721 PMC7926028 33455057

[65] Gkretsi, V. & Stylianopoulos, T. Cell adhesion and matrix stiffness: coordinating Cancer Cell Invasion and Metastasis. *Front. Oncol.* **8**, 145. 10.3389/fonc.2018.00145 (2018). 29780748 10.3389/fonc.2018.00145 PMC5945811

[66] Yang, P. et al. A large-scale retrospective study of the overall survival outcome in nasopharyngeal carcinoma with hypertension in Chinese population. *Oncotarget*. **8**, 75577–75586. 10.18632/oncotarget.17483 (2017). 29088892 10.18632/oncotarget.17483 PMC5650447

[67] Lengkey, R., Soetadji, R., Ardo & Sanjaya Use of angiotensin–converting enzyme inhibitors in gynecological cancers: pathways and mechanisms involved (review). *World Acad. Sci. J.* **6**(5). 10.3892/wasj.2024.263 (2024).

[68] Khan, N. A. et al. Unraveling the relationship between the renin-angiotensin system and endometrial cancer: a comprehensive review. *Front. Oncol.* **13**, 1235418. 10.3389/fonc.2023.1235418 (2023). 37869088 10.3389/fonc.2023.1235418 PMC10585148

[69] Michalczyk, K. & Cymbaluk-Płoska, A. Metalloproteinases in Endometrial Cancer-Are they worth measuring? *Int. J. Mol. Sci.* **22**(22), 12472. 10.3390/ijms222212472 (2021). 34830354 10.3390/ijms222212472 PMC8624741

[70] Duco, M. R., Murdock, J. L. & Reeves, D. J. Vascular endothelial growth factor inhibitor induced hypertension: retrospective analysis of the impact of blood pressure elevations on outcomes. *J. Oncol. Pharm. Pract.* **28**(2), 265–273. 10.1177/1078155220985915 (2022). 33430688 10.1177/1078155220985915

[71] Lofterød, T. et al. Exploring the effects of lifestyle on breast cancer risk, age at diagnosis, and survival: the EBBA-Life study. *Breast Cancer Res. Treat.* **182**, 215–227. 10.1007/s10549-020-05679-2 (2020). 32436147 10.1007/s10549-020-05679-2 PMC7275030

[72] Belo, V. A., Guimarães, D. A. & Castro, M. M. Matrix metalloproteinase 2 as a potential mediator of vascular smooth muscle cell Migration and Chronic Vascular Remodeling in Hypertension. *J. Vasc Res.* **52**(4), 221–231. 10.1159/000441621 (2015). 26731549 10.1159/000441621

[73] Copland, E. et al. Antihypertensive treatment and risk of cancer: an individual partici-pant data meta-analysis. *Lancet Oncol.* **22**(4), 558–570. 10.1016/S1470-2045(21)00033-4 (2021). 33794209 10.1016/S1470-2045(21)00033-4 PMC8024901

[74] Carlos-Escalante, J. A. et al. The use of antihypertensive drugs as Coadjuvant Therapy in Cancer. *Front. Oncol.* **11**, 660943. 10.3389/fonc.2021.660943 (2021). 34094953 10.3389/fonc.2021.660943 PMC8173186

[75] Mravec, B., Horvathova, L. & Hunakova, L. Neurobiology of Cancer: the role of β-Adrenergic receptor signaling in various tumor environments. *Int. J. Mol. Sci.* **21**(21), 1–24. 10.3390/ijms21217958 (2020). 10.3390/ijms21217958 PMC7662752 33114769

[76] Nguyen, N. T. H. et al. Renin-angiotensin-aldosterone system inhibitors and development of gynecologic cancers: a 23 million Individual Population-based study. *Int. J. Mol. Sci.* **24**(4), 3814. 10.3390/ijms24043814 (2023). 36835224 10.3390/ijms24043814 PMC9968233

[77] Pati, S., Irfan, W., Jameel, A., Ahmed, S. & Shahid, R. K. Obesity and Cancer: a current overview of Epidemiology, Pathogenesis, outcomes, and management. *Cancers*. **15**(2), 485. 10.3390/cancers15020485 (2023). 36672434 10.3390/cancers15020485 PMC9857053

[78] Davis, S. R. et al. *Menopause Nat. Rev. Dis. Primers* ;**1**, 15004. (2015). 27188659 10.1038/nrdp.2015.4

[79] Onstad, M. A., Schmandt, R. E. & Lu, K. H. Addressing the role of obesity in Endometrial Cancer Risk, Prevention, and treatment. *J. Clin. Oncol.* **34**(35), 4225–4230. 10.1200/JCO.2016.69.4638 (2016). 27903150 10.1200/JCO.2016.69.4638 PMC5455320

[80] Wang, Y., Zeng, X., Tan, J., Xu, Y. & Yi, C. Diabetes mellitus and endometrial carcinoma: risk factors and etiological links. *Med. (Baltim).* **101**(34), e30299. 10.1097/MD.0000000000030299 (2022). 10.1097/MD.0000000000030299 PMC9410665 36042597

[81] Saewai, C., Fumaneeshoat, O., Thongsuksai, P. & Ingviya, T. Diabetes Mellitus as Cancer Risk: a 14-year, cross-sectional analysis. *Nutr. Cancer*. **75**(6), 1454–1463. 10.1080/01635581.2023.2205054 (2023). 37099762 10.1080/01635581.2023.2205054

[82] Suh, S. & Kim, K. W. Diabetes and cancer: cancer should be screened in routine diabetes assessment. *Diabetes Metab. J.* **43**, 733–743. 10.4093/dmj.2019.0177 (2019). 31902143 10.4093/dmj.2019.0177 PMC6943263

[83] Fuhrman, B. J. et al. Association of the age at Menarche with Site-Specific Cancer risks in Pooled Data from nine cohorts. *Cancer Res.* **81**(8), 2246–2255. 10.1158/0008-5472.CAN-19-3093 (2021). 33820799 10.1158/0008-5472.CAN-19-3093 PMC8137527

[84] Londero, A. P., Parisi, N., Tassi, A., Bertozzi, S. & Cagnacci, A. Hormone replacement therapy in Endometrial Cancer survivors: a Meta-analysis. *J. Clin. Med.* **10**(14), 3165. 10.3390/jcm10143165 (2021). 34300331 10.3390/jcm10143165 PMC8303659

[85] Mutlu, L. et al. Endometrial Cancer in Reproductive Age: Fertility-Sparing Approach and Reproductive outcomes. *Cancers*. **14**(21), 5187. 10.3390/cancers14215187 (2022). 36358604 10.3390/cancers14215187 PMC9656291

[86] Burchardt, N. A., Shafrir, A. L., Kaaks, R., Tworoger, S. S. & Fortner, R. T. Oral contraceptive use by formulation and endometrial cancer risk among women born in 1947–1964: the nurses’ Health Study II, a prospective cohort study. *Eur. J. Epidemiol.* **36**(8), 827–839. 10.1007/s10654-020-00705-5 (2021). 33331993 10.1007/s10654-020-00705-5 PMC8416825

[87] Dimou, N. et al. Cigarette Smoking and Endometrial Cancer Risk: Observational and Mendelian Randomization Analyses. Cancer epidemiology, biomarkers & prevention: a publication of the American Association for Cancer Research, cosponsored by the American Society of Preventive Oncology, **31**(9), 1839–1848. 10.1158/1055-9965.EPI-21-1176 (2022). 10.1158/1055-9965.EPI-21-1176 PMC9437565 35900194

[88] Surendran, P. et al. Trans-ancestry meta-analyses identify rare and common variants associated with blood pressure and hypertension. *Nat. Genet.* **48**, 1151–1161 (2016). 27618447 10.1038/ng.3654 PMC5056636

